# Are Stripes Beneficial? Dazzle Camouflage Influences Perceived Speed and Hit Rates

**DOI:** 10.1371/journal.pone.0061173

**Published:** 2013-04-24

**Authors:** Bettina von Helversen, Lael J. Schooler, Uwe Czienskowski

**Affiliations:** 1 Max Planck Institute for Human Development, Center for Adaptive Behavior and Cognition, Berlin, Germany; 2 Department of Psychology, University of Basel, Basel, Switzerland; University of Sussex, United Kingdom

## Abstract

In the animal kingdom, camouflage refers to patterns that help potential prey avoid detection. Mostly camouflage is thought of as helping prey blend in with their background. In contrast, disruptive or dazzle patterns protect moving targets and have been suggested as an evolutionary force in shaping the dorsal patterns of animals. Dazzle patterns, such as stripes and zigzags, are thought to reduce the probability with which moving prey will be captured by impairing predators' perception of speed. We investigated how different patterns of stripes (longitudinal—i.e., parallel to movement direction–and vertical–i.e., perpendicular to movement direction) affect the probability with which humans can hit moving objects and if differences in hitting probability are caused by a misperception of speed. A first experiment showed that longitudinally striped objects were hit more often than unicolored objects. However, vertically striped objects did not differ from unicolored objects. A second study examining the link between perceived speed and hitting probability showed that longitudinally and vertically striped objects were both perceived as moving faster and were hit more often than unicolored objects. In sum, our results provide evidence that striped patterns disrupt the perception of speed, which in turn influences how often objects are hit. However, the magnitude and the direction of the effects depend on additional factors such as speed and the task setup.

## Introduction

The concept of camouflage refers to the ability of patterns to impair the chances that a target will be caught. Camouflage usually applies to stationary objects. In contrast, the concept of dazzle has been introduced to describe how disruptive patterns can protect moving targets in the same way. Dazzle has mostly been studied in the animal kingdom; for instance, researchers have investigated the impact of dorsal patterns on escape chances in snakes [1]–[5]. But dazzle can also influence human perception. In both world wars, navies painted their warships with high-contrast patterns in the hope of confusing the enemy [6]. There is no direct evidence that the dazzle designs indeed protected the ships, but recent research has demonstrated that dazzle patterns can influence the ability of humans to hit moving objects [7]. The mechanisms, however, with which dazzle patterns induce higher escape rates are not yet well understood. In general, it is thought that patterns impair the ability to accurately perceive speed, which in turn decreases the ability to capture objects [1]–[3], [7], [8]. The idea that objects with dazzle patterns disrupt the perception of speed is supported by a study showing that objects with disruptive patterns, such as zigzags or checks, were perceived as moving more slowly than unicolored objects [9].

Although the assumption that a misperception of speed should affect the ability to hit a moving target is intuitively appealing, some psychological research suggests that perceived speed plays only a minor role when hitting moving targets [10], [11]. Studies have revealed that perception and action are often based on different kinds of information [12]–[14]. Furthermore, a computerized experiment that manipulated perceived speed without affecting the object's position on the screen [10] found that perceived speed did not affect the trajectory of the hand movement to catch the object. Thus, even though dazzle patterns have been found to influence the ability to hit a moving object and to influence perceived speed, the effect of pattern on hitting probability may not be directly linked to the effect of dazzle patterns on perceived speed.

Our main goals were (1) to investigate if dazzle patterns influence (a) the ability to hit moving targets and (b) the perceived speed of the objects and (2) to test if perceived speed is related to hitting probability. A secondary goal was to investigate how specific dazzle patterns, that is, longitudinal and vertical stripes, influence the probability of hitting a moving target and perceived speed. Researchers largely agree that stripes can influence the perception of speed, but it is less clear if the orientation of the stripes affects whether objects are perceived as moving faster or slower (see [1], [5], [8], [15]). Stripes can be oriented either parallel or perpendicular to movement direction. In the following, we refer to stripes parallel to movement direction as longitudinal stripes, and stripes perpendicular to movement direction as vertical stripes. It has been debated if longitudinal stripes increase or decrease perceived speed and if longitudinally striped objects are perceived as faster than vertically striped objects. On the one hand, Gabor patches aligned with the direction of movement were perceived as moving faster than patches set at an angle to the direction of movement [16], suggesting that longitudinal patterns may be perceived as faster than vertical stripes (see also [15]). On the other hand, it has been argued that longitudinal stripes may have an advantage over vertical stripes in straight, high-speed flight because they make a snake appear to move more slowly than it actually does [1]–[3]. In addition, vertical stripes–although they have good cryptic properties if the snake stays still–are thought to provide reference points [1] that enhance movement detection and speed estimation [5]. In support of this theory, studies [1], [3] have shown a genetic correlation between flight behavior and dorsal patterns in garter snakes. In contrast, others did not find a difference in how easily vertically and longitudinally striped objects were hit [7], [8] or in perceived speed [9].

To explore the influence of dazzle patterns, we conducted two experiments. In Experiment 1 we investigated how the orientation of stripes (longitudinal vs. vertical) influences the probability with which moving targets are hit. In Experiment 2 we focused on the effect of pattern on perceived speed and the link between perceived speed and hit rate.

## Experiment 1

**Figure 1:**
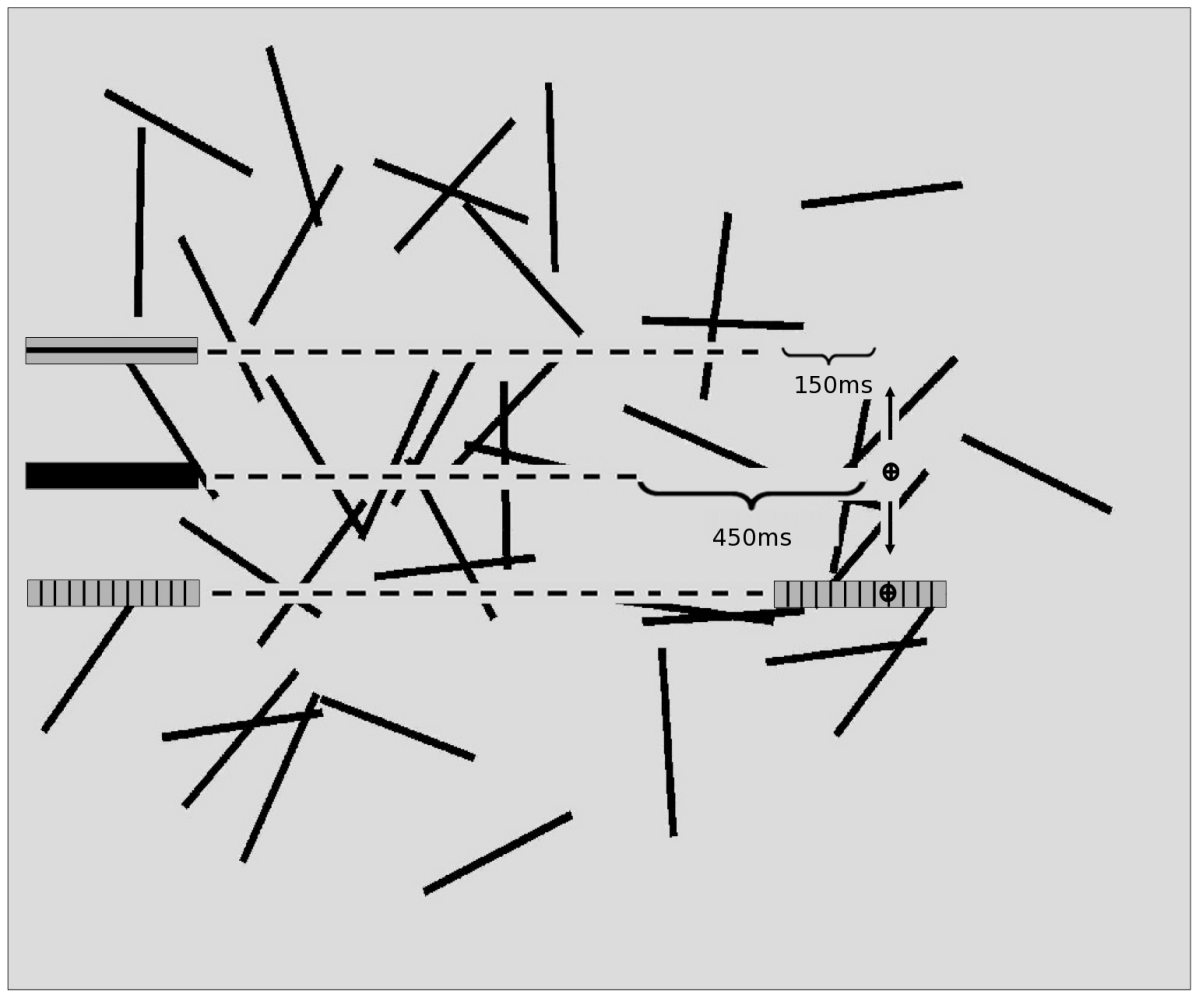
Experimental layout. The longitudinally striped, unicolored, and vertically striped objects are shown on the left. During the experiment, objects appeared on the left side of the screen at varying heights. The cursor (black circle) marks the position of the cursor at the beginning of a trial. The arrows above and below indicate the direction of the cursor movement. The dotted lines indicate possible paths of the objects and where they can be intercepted with the cursor. The brackets indicate the duration of the time interval during which objects would disappear.

In Experiment 1 we investigated the ability of humans to hit unicolored and striped objects in a computer game. In the game, objects moved from left to right across the screen in a straight line, starting at different heights. Objects could be hit by moving a cursor over the object with a joystick and pressing the fire button while the object was under the cursor. The cursor was positioned at ¾ of the width of the screen and could only be moved vertically (see Figure 1). Thus the task was to move the cursor to the vertical location of the object on the screen and to press the fire button when the object reached the target zone. We used three different surface patterns for the objects: unicolored, longitudinally striped, and vertically striped. If dazzle patterns have an advantage, the striped objects should be hit less often than the unicolored objects. Furthermore, if stripes differ in their effect on perceived speed and hit rates, and longitudinal stripes have an advantage over vertical stripes in straight flight, longitudinally striped objects should be hit less often than vertically striped objects.

Besides pattern, we varied the speed with which objects moved and a time interval (disappearance duration) during which objects were not visible before they had to be hit. We varied objects' speed so we could test if the effect of pattern is robust across different speeds. We introduced the disappearance duration because in natural conditions, a snake, for instance, may vanish in the grass and out of the field of view of a predator. In addition, having the objects disappear makes it more difficult for participants to rely on an object's current position [17], which should encourage them to rely on their perception of the object's speed.

### Methods

#### Ethics Statement

The study was conducted in accordance with the Declaration of Helsinki and approved by the ethics committee of the Max Planck Institute for Human Development in Berlin. All participants were blind to the hypotheses of the experiments and participated voluntarily. Written informed consent was received from all participants.

#### Participants

Fifty-eight students from Berlin universities participated in Experiment 1. Thirty were male; mean age was 24.7 years (*SD* = 2.76). The experiment took about 30 min and participants received €4 for participating. Depending on performance, they earned an additional €3 on average.

#### Design, Procedure, and Material

In Experiment 1, the participants' task was to hit objects moving from left to right on a computer screen with a cursor controlled by a joystick. We varied the pattern of the objects, pitting longitudinally striped and vertically striped against unicolored black objects. To hit an object, participants had to move the cursor over the object and press the fire button. The cursor was positioned at ¾ of the width of the screen and could only be moved vertically (see Figure 1). Because objects disappeared shortly before they reached the point where they could be hit (see [11] for a similar design), participants had to press the fire button when they thought the object would be under the cursor without seeing the object. Objects reappeared after participants pressed the trigger. If participants fired while the cursor was over the object it counted as a hit, and otherwise a miss was recorded.

The experiment was conducted in the laboratory of the Max Planck Institute for Human Development. The room was artificially lit (standard fluorescent laboratory lighting) and lighting was kept approximately constant for all participants. The experiment was conducted on two computers, both with Microsoft Sidewinder joysticks and with 17-inch CRTs with refresh rates of 85 Hz and a resolution of 1,024×768 pixels and default brightness and contrast settings. Participants were seated in front of the screen, with their heads approximately 60 cm from the screen. The experimental software was implemented using the C# language under Microsoft.NET 2.0 with Managed DirectX. The screen background was stylized “grass,” light grey (color code in the red, green, blue [RGB] color model: 220, 220, 220) with thirty 40 mm×2 mm (3.82×0.19 degrees of visual angle) black lines (RGB color code: 0, 0, 0). The angle, orientation, and location of the lines were randomly determined. We created 20 different backgrounds. For each participant we randomly selected one background for the game. Objects were rectangles of 39 mm length and 7 mm width (3.72×0.67 degrees of visual angle). Eighty percent of the surface of the two patterned objects was medium grey (RGB color code: 180, 180, 180) and the remaining 20% was black (RGB color code: 0,0,0). The longitudinally striped object had one black stripe running across the middle (1.4 mm×39 mm, 0.13×3.72 degrees of visual angle). The vertically striped object had 11 black stripes (0.7 mm×7 mm, 0.07×0.67 degrees of visual angle) equidistantly distributed over the object (see Figure 1). The unicolored object was black (RGB color code: 0,0,0).

In addition to object pattern we varied the objects' speed and the duration of the time during which objects disappeared from sight. Objects were either slow (12 cm/s, 11.42 degrees of visual angle per second [deg/s] given the distance from the screen) or fast (16 cm/s, 15.19 deg/s). We selected these speeds based on a pilot. In the pilot we used an adaptive testing procedure to determine the speed at which participants hit unicolored objects with a probability of 0.75 [18]. The pilot was similar to the task used in Experiment 1, but objects did not disappear from the screen. We then selected speeds in the medium and lower portion of the speed distribution that were comparable to speeds used in the literature [11].

In the experimental task the objects disappeared before they could be hit. The *disappearance duration* specified the time objects needed to travel from the point they vanished to the point where they could be hit. We varied the disappearance duration in two conditions: long: 450 ms vs. short: 150 ms. This resulted in a Speed (slow vs. fast) × Disappearance Duration (short vs. long) × Pattern (longitudinally striped, vertically striped, unicolored) within-subject design. Pattern, speed and disappearance duration were varied randomly from trial to trial.

The experiment consisted of a training phase of 50 trials and a test phase of 360 trials, with 30 trials in each condition. During training participants practiced with unicolored black objects to familiarize themselves with the task. In the training phase, to obtain an average hit rate of 50%, object speed was set by an adaptive step algorithm [18], i.e. if the object was hit, the object in the next trial moved faster, but if an object was missed, the object in the next trial moved slower.

Participants received visual and auditory feedback. If the object was hit, a tone sounded, the object turned red, and the trial ended. If it was missed, it continued on its path until it vanished off the right side of the screen. Participants were given one shot per trial. After each trial the cursor returned to the starting position and could not be moved until the next trial was started with the space bar. To reduce error variance, 80% of the objects appeared in the two middle quarters of the screen and 20% fell into the two extreme quarters.

### Results and Discussion

**Figure 2:**
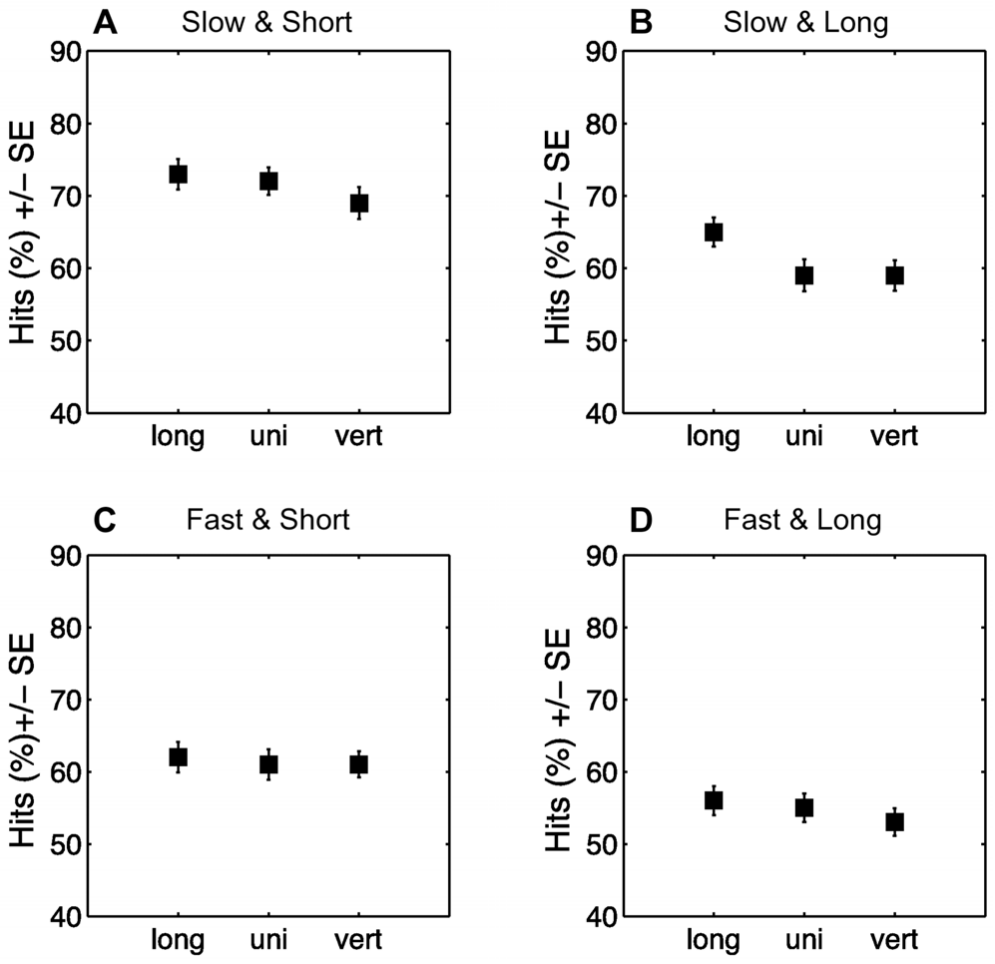
Mean percentages of hit rate by pattern, speed, and disappearance duration. (A) Slow speed and a short disappearance duration. (B) Slow speed and long disappearance duration. (C) Fast speed and short disappearance duration. (D) Fast speed and long disappearance duration. Vert  =  vertically striped object; long  =  longitudinally striped object, uni  =  unicolored object. Error bars denote the standard error.

Experiment 1 varied the pattern of the objects, the objects' speed, and the time interval between when the objects disappeared and when they could be hit (disappearance duration). We analyzed whether pattern influenced the frequency with which the objects were hit with a repeated measurement analysis of variance (ANOVA) with percentage of hits as the dependent variable and pattern, speed, and disappearance duration as within-subject factors. To minimize variance, we focused on the 288 experimental trials that were located in the middle quarters of the screen (24 trials per condition). We then calculated contrasts comparing the longitudinally striped and the vertically striped pattern with unicolored objects (see Figure 2).

Overall, we found that participants hit objects on average in 62% of the trials. Objects that disappeared for 450 ms were hit less often than objects that disappeared for 150 ms (ANOVA, *F*_1,57_ = 93.3, *P*<0.001), and fast objects were hit less often than slow ones (ANOVA, *F*_1,57_ = 4.78, *P* = 0.03). The effect of speed was larger with a short disappearance duration than with a long one, as indicated by a significant interaction between speed and disappearance duration (ANOVA, *F*_1,57_ = 4.78, *P* = 0.03).

More importantly, we found a significant main effect of pattern (ANOVA, *F*_2,114_ = 6.18, *P* = 0.003). Contrasts showed that longitudinally striped objects were hit more often than unicolored objects (Contrast, *F*_1,57_ = 5.63, *P* = 0.02), but vertically striped objects were not statistically significantly different from unicolored ones (Contrast, *F*_1,57_ = 1.64, *P* = 0.21). None of the interactions–between speed and pattern (ANOVA, *F*_2,114_ = 2.42, *P* = 0.09), between disappearance duration and pattern (ANOVA, *F*_2,114_ = 1.56, *P* = 0.21), or between pattern, speed, and disappearance duration (ANOVA, *F*_2,114_ = 2.18, *P* = 0.12) –reached significance. As illustrated in Figure 2, separate follow-up tests for speed and disappearance duration, however, found a significant effect of pattern with a long disappearance duration and slow speed (ANOVA, *F*_2,114_ = 9.56, *P*<0.001; see Figure 2B) and with a short disappearance duration and slow speed (ANOVA, *F*_2,114_ = 3.20, *P* = 0.04, see Figure 2A). The effects of pattern were nonsignificant when objects moved at high speed (Figure 2C: short disappearance duration and high speed, ANOVA, *F*_2,114_ = 0.05, *P* = 0.95; Figure 2D: long disappearance duration and high speed, ANOVA, *F*_2,114_ = 1.30, *P* = 0.28).

In sum, we found that objects were hit less frequently when they moved faster and when they disappeared for longer. These results are not surprising, as both factors increase the difficulty of the task. More importantly, the experiment showed a significant effect of pattern on hit rates. In contrast to the dazzle hypothesis, however, longitudinally striped objects were hit more often than vertically striped or unicolored ones. These results resonate with research reporting that dazzle patterns influence the probability with which moving objects can be hit [1], [7] but suggest that the effect need not be beneficial. We will discuss this point in the General Discussion.

Overall, the effect of pattern on hitting probability seemed reliable, as we did not find a significant interaction between pattern and disappearance duration or pattern and speed. Follow-up tests, however, did not find a difference between objects' patterns for fast-moving objects, suggesting that the effect of pattern may be limited to lower speeds. The effect of pattern could diminish when objects move fast, because at high speeds the perception of speed may be less reliable in general, masking effects caused by pattern. Furthermore, at high speeds vertical stripes can blend together, giving the impression of a unicolored object and eliminating effects of vertical stripes. This blurring is known as the flicker fusion phenomenon [19]–[22].

To replicate the effects found in Experiment 1, we conducted a further experiment (Experiment 1.1) with the same experimental setup but with a reduced short disappearance duration. The results showed a similar pattern to that of Experiment 1. Details for Experiment 1.1 can be found in the supplementary online material, Experiment S1.

## Experiment 2

In Experiment 1 we investigated the effect of pattern on the probability with which objects can be hit, but we did not measure perceived speed. To clarify the relationship between perceived speed and hitting probability we conducted a second experiment where we measured perceived speed and hit rates in the same experiment. Additionally, we simplified the hitting task such that all objects appeared at the same vertical position on the screen, eliminating the need to adjust the cursor to the correct vertical position. Having to move the cursor could be a potential source of error that could mask the effects of perceived speed, particularly because pattern may influence other aspects of stimulus perception [23], [24].

### Methods

#### Ethics Statement

The study was conducted in accordance with the Declaration of Helsinki and approved by the ethics committee of the Max Planck Institute for Human Development in Berlin. All participants were blind to the hypotheses of the experiments and participated voluntarily. Written informed consent was received from all participants.

#### Participants

Thirty students from Berlin universities participated in Experiment 2. Seventeen were female, with a mean age of 25.23 years (*SD* = 3.23). Participants received on average €7 for their participation. We excluded one participant from the analysis because she performed more than three standard deviations below the mean, but the pattern of results does not change if she is included.

#### Design and Procedure

The study consisted of two parts: a perceptual task with the goal of comparing the speed of two objects and a motor task with the goal of hitting the objects. The order of the tasks was randomized with half of the participants beginning with the speed comparison task and half with the hitting task. The same materials as in Experiment 1 were used and the experiment was conducted in the same laboratory and under similar lighting conditions, kept approximately constant between participants. However, we used the same background for all participants and the experiment was run on a computer with a 17-inch LCD screen with a resolution of 1,280×1,024 pixel. Brightness and contrast were set at default values. This resulted in a displayed size of the objects of 31 mm length×5 mm width (2.96×0.48 degrees of visual angle). We measured luminance with a luminance meter (Gossen Mavo-Monitor, Germany). The luminance values were (in cd m^−2^): Black = 0.4, medium grey (objects' stripes) = 75, light grey (background) = 125. Unfortunately we cannot provide luminance measures for the first experiment, because the monitors used in this experiment had been replaced before we acquired a luminance meter.

#### Speed Comparison

In the speed comparison task participants saw two objects moving one after the other across the screen. After both objects had vanished, participants indicated if the second object was slower, equally fast, or faster than the first object. Each comparison consisted of a target object and a comparison object. The comparison object was a unicolored black object that moved with a speed of 12 cm/s (11.42 deg/s). The target object was unicolored or striped vertically or longitudinally. Whether the target or the comparison object was seen first was randomly determined from trial to trial. The speed of the target object was adjusted using an adaptive staircase algorithm [18]. If the participant indicated that the target object had been faster than the comparison object, the target object's speed was reduced in the next trial. If the participant indicated that the target object had been slower than the comparison object, the target object's speed was increased in the next trial. If the participant indicated the objects moved at equal speed, the target object's speed was decreased or increased depending on the direction of the last speed change. For example, if the speed had been reduced in the trial before and the participant now indicated that the speed was equal, the target object's speed was further reduced in the subsequent trials until the participant indicated that it was now slower than the comparison object. The speed comparison in each staircase continued until six reversals in speed adjustment (i.e., the speed of the target object was increased in one trial and decreased in the next trial) took place. In the first rounds the speed was adjusted by 1 cm/s (0.95 deg/s). After three reversals it was adjusted by 0.5 cm/s (0.48 deg/s). For each target object we had four staircases with different starting speeds: 8, 10, 14, and 16 cm/s (7.62, 9.53, 13.31, and 15.19 deg/s). Pattern and staircase were varied randomly from trial to trial.

#### Hitting Task

The hitting task was similar to the setup in Experiment 1 (see Figure 1). After a short practice period, participants played 180 trials of a hitting task. Objects appeared on the left side of the screen and moved at a constant speed of 12 cm/s (11.42 deg/s) in a direct line across the screen. The participants' task was to fire when objects passed under the cursor position, but similar to Experiment 1, the objects disappeared shortly before they reached the cursor location. In contrast to Experiment 1, the objects always appeared at the same vertical location. Thus in this version of the hitting task, it was not necessary to adjust the cursor position and no joystick was required. Instead participants were instructed to fire by pressing the return key when they thought the object was located under the cursor. We varied the pattern of the object and the disappearance duration, resulting in a within-subject design with two factors. Disappearance duration varied with two levels, 350 ms and 150 ms. We reduced the long disappearance duration in comparison to Experiment 1 to investigate if the effect was reliable at different disappearance durations and to ensure that the objects were visible long enough to allow participants to form an accurate perception of speed. There were three types of patterns: the objects were unicolored, vertically striped, or longitudinally striped (see Figure 1). In each condition participants performed 30 trials. The pattern and disappearance duration were varied randomly from trial to trial. To reduce variance in where participants tried to hit the objects, we instructed participants to aim for the middle of the object and paid them depending on performance. They received 5 points for hitting the center (the area less than 2.5 mm/0.24 degrees of visual angles from the center), 2 points for hitting close to the center (the areas more than 2. 5 mm/0.24 degrees of visual angles and less than 9 mm/0.86 degrees of visual angles from the center), and 1 point for hitting the object at the front or the back (the area close to the front or back and more than 9 mm/0.86 degrees of visual angles from the center).

### Results and Discussion

**Figure 3:**
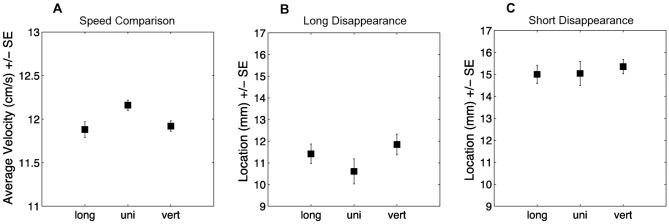
(A) Average perceived speed of unicolored and striped objects. (B) Average location of the object in the long disappearance condition. (C) Average location of the object in the short disappearance condition. Vert  =  vertically striped object; long  =  longitudinally striped object, uni  =  unicolored object. Error bars denote a standard error.

To obtain an estimate of perceived speed, we took the average speed in the trials where participants had responded that the target object moved with the same speed as the unicolored comparison object for each pattern separately. We analyzed the data with a repeated measurement ANOVA with pattern as within-subject factor and perceived speed as dependent variable. We additionally calculated contrasts comparing striped objects with unicolored objects. As illustrated in Figure 3A, the results showed that for both vertically striped (Contrast, *F*_1,28_ = 6.86, *P* = 0.02) and longitudinally striped (Contrast, *F*_1,28_ = 9.96, *P* = 0.005) objects the average speed at which they were perceived as moving equally fast as the comparison object was slower than for the unicolored objects. This suggests that at the same speed, the striped objects were perceived as moving faster than unicolored objects.

As a measure of hitting success, we measured the frequency with which participants hit the objects (for means and standard deviations see Table 1). We analyzed the data with a repeated measurement ANOVA with pattern and disappearance duration as within-subject factors and hit rate as dependent variable. Objects that disappeared for 350 ms were hit less often than objects disappearing for 150 ms (ANOVA, *F*_1,28_ = 25.91, *P*<0.001). There was no main effect of pattern on hit rates (ANOVA, *F*_2,56_ = 1.56, *P* = 0.22), but we found a significant interaction between disappearance duration and pattern (ANOVA, *F*_2,56_ = 4.72, *P* = 0.01). Follow-up analyses for both disappearance durations separately revealed that when objects disappeared for 350 ms, unicolored objects were hit significantly less than longitudinally striped objects (Contrast, *F*_1,28_ = 4.44, *P* = 0.04) and vertically striped objects (Contrast, *F*_1,28_ = 6.39, *P* = 0.02). There was no effect with a short disappearance duration (ANOVA, *F*_2,56_ = 1.39, *P* = 0.26).

**Table 1:** Means and Standard Deviations of Hit Rate by Disappearance Duration and Pattern.

	Disappearance duration					
	150 ms			350 ms		
	Pattern			Pattern		
	Long	Uni	Vert	Long	Uni	Vert
Hit rate	0.97 (0.05)	0.98 (0.03)	0.98 (0.04)	0.96 (0.04)	0.94 (0.05)	0.96 (0.04)

*Note*. *N* = 29. Hit rate gives the percentage of hits. Long: longitudinally striped; uni: unicolored; vert: vertically striped.

Additionally we measured where the object was, when participants fired (i.e. pressed the return button), with a location of zero corresponding to the front of the object being at the cursors location (i.e. the object would be hit close to the front) and a location of 31 mm corresponding to the back of the object being at the cursors location (i.e. the object was hit close to the back, see Figure 3). We analyzed objects' location with a repeated measurement ANOVA with location as dependent variable and pattern and disappearance duration as independent variables. The analysis of the data showed that all objects had moved along less (i.e. were hit closer to the front) with a long disappearance duration than with a short disappearance duration (ANOVA, *F*_1,28_ = 197.6, *P*<0.001), but there was no main effect of pattern (ANOVA, Greenhouse–Geisser corrected, *F*_1.52,42.64_ = 2.20, *P* = 0.14). Separate ANOVAs for a short and a long disappearance duration indicated that pattern influenced objects' location if the disappearance duration was long (ANOVA, *F*_2,56_ = 3.79, *P* = 0.03). As illustrated in Figure 3 (middle panel), unicolored objects were hit closer to the front than vertically striped objects (Contrast, *F*_1,28_ = 6.70, *P* = 0.02), but not closer to the front than longitudinally striped objects (Contrast, *F*_1,28_ = 2.43, *P* = 0.13). We did not find an effect of pattern with a short disappearance duration (ANOVA, *F*_2,56_ = 0.33, *P* = 0.72; see Figure 3 right panel).

Overall, the results suggest that pattern had an influence on the objects perceived speed, the objects' location when participants fired, and on the frequency with which they were hit. To test if the effect of perceived speed was related to the location of the hit, we correlated the difference in perceived speed with the location where the striped objects were hit. We found that the faster vertically striped objects were perceived to be moving compared to unicolored objects, the more to the back they were hit in the long disappearance duration condition (*N* = 29, Pearson correlation, *r* = 0.44, *P* = 0.02). The location where longitudinally striped objects were hit did not significantly correlate with perceived speed (*N* = 29, Pearson correlation, *r* = 0.16, *P* = 0.42).

In sum, Experiment 2 showed that pattern had an influence on perceived speed, the probability with which objects were hit, and the objects' location when participants fired. Participants perceived striped objects that moved slower than unicolored objects as equally fast and hit striped objects more to the back compared to unicolored objects. Furthermore, we found a correlation between the degree with which people misperceived the speed of vertically striped objects and the location of the hit, providing further support that stripes influence the perception of speed, which in turn influences how the objects are hit. This supports the idea that dazzle patterns can affect the ability to hit moving targets and that this effect is caused by a misperception of speed, resonating with earlier research [7]–[9]. Somewhat surprisingly, striped objects were hit more to the back than unicolored objects, although they were perceived as moving faster. If objects move slower than perceived one would expect more hits to the front of the object. The correlation between differences in perceived speed and the objects' location, however, suggests that the effect was related to speed perception. One possible explanation is that dazzle patterns decrease confidence in speed perception, which in turn decreased reaction times.

Similar to in Experiment 1, where the effect of pattern was stronger when objects disappeared for 450 ms than when they disappeared for 150 ms, the effect of pattern appeared only if the objects vanished for 350 ms in the “grass.” This suggests that pattern may influence hit rate only when objects are out of sight before they can be hit. The effect of pattern could be stronger with longer disappearance durations, because the disappearance of the objects forced participants to rely on perceived speed to estimate when the object would reach the target zone. When objects do not disappear or disappear for only a short time, people may instead use the objects' position to decide when to fire, which in turn could reduce the influence of pattern on hit rates. This explanation resonates with research suggesting that people strongly rely on position when hitting moving targets [10]–[11].

## General Discussion

### The Influence of Dazzle Patterns on Perceived Speed and Hit Rate

In two experiments we demonstrated that dazzle patterns influence the perception of speed, which in turn influences the rate at which moving targets are hit. In both experiments, longitudinally striped objects were hit more often than unicolored objects; and in Experiment 2, but not in Experiment 1, vertically striped objects were hit more often than unicolored objects. In addition, Experiment 2 offered direct evidence that striped objects were perceived as moving faster than unicolored objects. These results are consistent with the observation that longitudinally striped snakes make the perception of speed more difficult [1] and with prior research reporting effects of dazzle patterns on hit rates [7]–[8] and perceived speed [9]. In contrast to [1] and [9] our results, however, suggest that striped patterns can be perceived as moving faster than unicolored objects. This suggests that the relation between perceived speed and dazzle pattern may be more complex and could depend on factors such as the type of comparison object, which was white in the study by [9], but black in our study.

The effect of pattern, however, differed somewhat between the two experiments. In Experiment 1, we did not find an effect of vertical stripes, but we did find an effect in Experiment 2. These differences could be due to chance, but also differences in the task setup of the experiments could have contributed. In Experiment 1 participants not only had to press the fire button at the right time (i.e., when they believed the object reached the horizontal position of the cursor), but they also had to adjust the cursor to the correct vertical position. In Experiment 2 the vertical position of the cursor was fixed. If vertical stripes impeded the perception of the object's vertical position [24], this could have reduced the hit rate for vertically striped objects in the first experiment.

A possible limitation of our experiment is that the luminance and contrast of the unicolored object and the striped objects differed. The unicolored black object had a lower luminance and higher contrast than the striped objects (which both were 80% grey and only 20% black). Visual contrast can influence perceived speed [25]–[27] and thus differences in contrast could have contributed to the differences in perceived speed and hit rates. Speaking against this explanation is that low contrast patterns are usually found to be moving slower than high contrast pattern and a similar effect for high- and low-contrast patterns on hit rates was found [7]. Furthermore, the effect of pattern on perceived speed was found to be more pronounced with high-contrast patterns than with low-contrast patterns [9].

### Benefits of Dazzle Patterns

In our experiments we found that dazzle patterns influenced the perception of speed, and had an impact on the probability of hitting the objects. However, in both experiments striped objects were hit more often than or equally often as unicolored objects, casting doubt on the proposed general benefit of dazzle patterns. There are at least two possible explanations for why in our task striped objects were hit more often than unicolored objects, whereas other research has found that dazzle patterns reduce how frequently objects are hit. For one, the way objects are captured could influence the results. For instance, it has been argued [28] that a relation between dorsal pattern and flight behavior in lizards was an adaptation to predation from the land versus the air. In our experiments participants were required to hit objects by waiting until they reached the target zone. However, many predators may hunt their prey by chasing them, which could in turn influence how dazzle pattern affect the ability to hit moving prey.

Second, the effect of pattern on hit rates could depend on the type of movement. Research on snakes suggests that one type of camouflage works well when a snake follows a curved flight path and another when the snake follows a straight path. As even the straight path of a fleeing snake is more serpentine than the strictly linear movement path of the objects in our task, this interaction could figure into our results.

In sum, future tests of the effects of patterns on chances of escape should take into account the natural situation in which these markings are found (see also [29]). It may not be enough to determine whether a particular pattern causes a misperception of an object's speed. Rather, it is essential to consider how this misperception interacts with the habitat and how the object moves or is pursued.

## Conclusion

In our experiments we found reliable but small differences between striped objects and a unicolored object, which we could trace to a misperception of speed caused by dazzle patterns. Striped objects were hit about 2% more often than unicolored objects. Although this may sound like a negligible effect, from an evolutionary perspective an increase in survival rate of 3% can result in a strong selection pressure [30]. Furthermore, the differences between striped and unicolored objects observed in our experiments are comparable to the effect sizes found in previous research [9], where it was argued that a misperception of this magnitude could be sufficient to grant protection from a roving predator.

## Supporting Information

Experiment S1 Replication of Experiment 1.
(PDF)
