# Supplementary material for: Are Stripes Beneficial? Dazzle Camouflage Influences Perceived Speed and Hit Rates
Source: PLoS One. 2013 Apr 24;8(4):e61173. doi: 10.1371/journal.pone.0061173 (PMC3634837; doi:10.1371/journal.pone.0061173)
Supplement: Experiment S1 — Replication of Experiment 1. (PDF) [file pone.0061173.s001.pdf]

Experiment S1

Replication of Experiment 1

To replicate the effects found in Experiment 1, we conducted a further Experiment 1.1, using the same method as in Experiment 1 with the only change being that we reduced the short disappearance duration from 150 ms to 0 ms. Forty students from Berlin universities participated. Fourteen were male, with a mean age of 23.25 years ($SD = 2.77$). Participants received on average €7 for their participation.

Results

Overall, we replicated the results from Experiment 1. Participants reached a similar level of accuracy: On average, 60% of the objects were hit. Again, fast objects were hit less often than slow objects (ANOVA, $F_{1,39} = 46.17$, $P < 0.001$) and objects that disappeared for 450 ms were hit less often than objects that did not disappear (ANOVA, $F_{1,39} = 129.51$, $P < 0.001$). Further, the effect of speed was larger when objects did not disappear (ANOVA, $F_{1,39} = 5.03$, $P = 0.03$; for means and *SD*s see Table S1). We also found a main effect for pattern (ANOVA, $F_{2,78} = 3.83$, $P = 0.03$). Longitudinally striped objects were hit more often than vertically striped ones (Contrast, $F_{1,39} = 9.10$, $P = 0.004$; for means and *SD*s see Table A1), but not reliably more often than unicolored ones (Contrast, $F_{1,39} = 1.05$, $P = 0.31$). We did not find an interaction between pattern and disappearance duration (ANOVA, $F_{2,78} = 2.07$, $P = 0.13$).

Table S1

Means and Standard Deviations of Hit Rate by Speed, Disappearance Duration, and Pattern

	Disappearance duration					
	0 ms			450 ms		
	Pattern			Pattern		
Speed	Long	Uni	Vert	Long	Uni	Vert
12 cm/s	0.76 (0.15)	0.74 (0.18)	0. 70 (0.15)	0.58 (0.18)	0.54 (0.15)	0.56 (0.18)
16 cm/s	0.61 (0.18)	0.63 (0.15)	0.59 (0.16)	0.49 (0.14)	0.48 (0.16)	0.48 (0.15)

Note. Long: longitudinally striped; uni: unicolored; vert: vertically striped.
